# Empirical vs Bayesian approach for estimating haplotypes from genotypes of unrelated individuals

**DOI:** 10.1186/1471-2156-8-2

**Published:** 2007-01-29

**Authors:** Shuying Sue Li, Jacob Jen-Hao Cheng, Lue Ping Zhao

**Affiliations:** 1 Division of Public Health, Fred Hutchinson Cancer Research Center, Seattle, WA, USA; 2 Quality Indicator Project, Maryland Hospital Association, MD, USA

## Abstract

**Background:**

The completion of the HapMap project has stimulated further development of haplotype-based methodologies for disease associations. A key aspect of such development is the statistical inference of individual diplotypes from unphased genotypes. Several methodologies for inferring haplotypes have been developed, but they have not been evaluated extensively to determine which method not only performs well, but also can be easily incorporated in downstream haplotype-based association analyses. In this paper, we attempt to do so. Our evaluation was carried out by comparing the two leading Bayesian methods, implemented in PHASE and HAPLOTYPER, and the two leading empirical methods, implemented in PL-EM and HPlus. We used these methods to analyze real data, namely the dense genotypes on X-chromosome of 30 European and 30 African trios provided by the International HapMap Project, and simulated genotype data. Our conclusions are based on these analyses.

**Results:**

All programs performed very well on X-chromosome data, with an average similarity index of 0.99 and an average prediction rate of 0.99 for both European and African trios. On simulated data with approximation of coalescence, PHASE implementing the Bayesian method based on the coalescence approximation outperformed other programs on small sample sizes. When the sample size increased, other programs performed as well as PHASE. PL-EM and HPlus implementing empirical methods required much less running time than the programs implementing the Bayesian methods. They required only one hundredth or thousandth of the running time required by PHASE, particularly when analyzing large sample sizes and large umber of SNPs.

**Conclusion:**

For large sample sizes (hundreds or more), which most association studies require, the two empirical methods might be used since they infer the haplotypes as accurately as any Bayesian methods and can be incorporated easily into downstream haplotype-based analyses such as haplotype-association analyses.

## Background

The completion of the HapMap project has stimulated further interest in haplotype inference from un-phased single nucleotide polymorphism (SNP) genotypes [1]. Recent evidence indicates the human genome has hot spots and cold spots for recombination, and it is divided into multiple haplotype blocks, each of which has only a limited number of haplotypes [2-5]. Such haplotype block structure in the human genome suggests that haplotype-based methods may play an important role in genetic association studies [6,7]. Haplotypes can be generated experimentally by dissecting out single chromosomes and inserting the entire chromosome into a yeast artificial chromosome [8] or by using rodent-human hybrid techniques to physically separate two chromosomes [5]. However, both technologies are experimentally challenging and cost prohibitive for use in population research at this time. The most commonly used technologies generate un-phased SNP genotypes. One way to resolve individual haplotypes is via family data, which is expensive to collect [9]. Another option is to resolve individual haplotypes statistically. Clark's heuristic algorithm is probably among the first statistical methods for inferring haplotypes from genotypes of unrelated individuals [10].

Many maximum likelihood methods have been developed, and almost all share the same scientific objectives and likelihood framework. The fundamental difference among these methods is a prior assumption for the distribution of haplotypes: methods with prior assumption for distribution of haplotypes are referred to as Bayesian methods, and the methods without any prior assumption are called empirical methods.

Estimation-maximization (EM) algorithm, a maximum likelihood method, was first introduced to infer haplotypes from unrelated individuals [11-13], but those earlier works were computationally demanding when processing large number of SNPs. More recently Qin et al. [14] discussed a new strategy for estimating haplotype frequencies using the EM algorithm, which largely improved performance, especially when analyzing data with large numbers of SNPs. Li et al. [15] have applied the estimating equation (EE) technique and further improved the statistical and computational efficiency in the estimation of haplotype frequencies and their standard errors. Both EM and EE methods are empirical methods.

Stephens et al. were probably among the first groups to propose a model-based Bayesian method [16] under the assumption of coalescence of haplotypes. Later it was modified to improve statistical and computational efficiency [17,18]. Niu et al. [19] took a Bayesian approach but chose a Dirichlet distribution for haplotypes as their prior, and published a computational algorithm to handle a large number of SNPs, which was referred to as partition-ligation (PL).

Which method performs the best? Several papers have attempted to address this question and their conclusions are not without controversy [14,19-22]. These papers compared the performances of haplotyping methods based on a limited number of available haplotype data sets and some simulated data. Recently, Marroni et al. [23] used genotype data provided by Illumina and Affymetrix for Genetic Analysis Workshop 14. The data include genotypes of 104 mother-son pairs with Caucasian ancestry on 313 SNPs of the X-chromosome. Because males have only one copy of the X-chromosome, mother haplotypes can be resolved from their sons' genotypes. Instead of evaluating the performances of the methods for analyzing SNPs within haplotype blocks, Marroni et al. investigated the 14 series of unphased genotypes of 5 or 10 SNPs with different values of linkage disequilibrium (LD). In this paper, we used the dense genotypes on the X-chromosome of 30 European and 30 African trios provided by the International HapMap project. The X-chromosome was chosen because mother haplotypes can be unambiguously resolved from the genotypes of trios. Resolved haplotypes were divided into blocks using the Haploview program [24], providing abundant haplotype data sets. Among the identified haplotype blocks, we randomly selected 500 blocks to evaluate haplotyping method performances. To evaluate the performances of haplotype methods on the data with larger sample sizes, we conducted some simulation studies under different scenarios. In our first set of simulations, we generated haplotypes based on real data on the X-chromosome. In our second set of simulations, we generated haplotypes using Hudson's coalescent program [25] to investigate how much efficiency is gained by assuming coalescence prior in PHASE compared to empirical methods without assuming a prior. Programs used for comparisons are PHASE (version 2.1) [26] for the model-based Bayesian method [20], HAPLOTYPER [27] for the empirical Bayesian method [19], PL-EM [28] for the EM method [14], and HPlus [29] for the EE method [15]. Because the accurate estimation of haplotype frequencies and inference of individual haplotypes are both critical in assessing haplotype association with disease phenotypes [30-34], our comparisons focus on evaluating method performance from these two angles.

## Methods

### HapMap Trio Data

We used X-chromosome genotype data of 30 European and 30 African trios from the HapMap project [1]. With trio data, mother haplotypes can be resolved unambiguously from her offspring's and the father of her offspring's genotype data. The mother's two chromosomes are separated at each locus as transmitted or not transmitted to her child. If the child is male, the child's X-chromosome is transmitted from his mother, therefore mother's allele that matches the child's allele is the transmitted allele. If the child is female, one chromosome is from her mother and the other is from her father. Using the father's allele on the X-chromosome, we can deduce which allele is transmitted from the mother. Hence, 30 mother haplotype pairs are determined by sorting out transmitted alleles from untransmitted alleles, and these sixty (= 30 × 2) represent the true haplotypes, which are not readily obtainable for any autosome chromosomes.

Applying this procedure to the HapMap Phase II data (July 2005 release), we obtained phase-resolved X-chromosome SNP data. We further divided these SNPs into haplotype blocks using Haploview software [24], by specifying for the method described in [35], the parameters of confidence interval minima 0.8 and 0.5 for strong LD, upper confidence interval maximum 0.6 for strong recombination, the fraction of strong LD in informative comparisons to be at least 0.95, and excluding the SNPs with MAF less than 0.05. We chose the parameters to be less stringent than default values to get larger blocks that are still in high LD. The block identification was done separately for European and African mother haplotypes. Within each population, we randomly chose 500 haplotype blocks to compare haplotyping methods. Among the 500 European mother haplotype blocks, the number of SNPs ranges from 2 to 195 with mean of 13 and median of 7. Among the 500 African mother haplotype blocks, the number of SNPs ranges from 2 to 33 with mean of 5 and median of 3. It had been shown that African populations have shorter haplotype blocks than European populations [1].

### Notations

Consider a sample of *n *unrelated individuals from a study population. From each individual, we observe *q *SNP-genotypes on a specific region in the genome. Let g˜i
 MathType@MTEF@5@5@+=feaafiart1ev1aaatCvAUfKttLearuWrP9MDH5MBPbIqV92AaeXatLxBI9gBaebbnrfifHhDYfgasaacH8akY=wiFfYdH8Gipec8Eeeu0xXdbba9frFj0=OqFfea0dXdd9vqai=hGuQ8kuc9pgc9s8qqaq=dirpe0xb9q8qiLsFr0=vr0=vr0dc8meaabaqaciaacaGaaeqabaqabeGadaaakeaacuWGNbWzgaGhamaaBaaaleaacqWGPbqAaeqaaaaa@2FAF@ = (*g*_*i*1_, …, *g*_*iq*_) denote the *q*SNP-genotypes for the *i*th individual. When genotype *g*_*ij *_is heterozygous, the phase (parental origin of the two alleles) becomes ambiguous and has two solutions denoted by *p*_*ij *_. Let p˜i
 MathType@MTEF@5@5@+=feaafiart1ev1aaatCvAUfKttLearuWrP9MDH5MBPbIqV92AaeXatLxBI9gBaebbnrfifHhDYfgasaacH8akY=wiFfYdH8Gipec8Eeeu0xXdbba9frFj0=OqFfea0dXdd9vqai=hGuQ8kuc9pgc9s8qqaq=dirpe0xb9q8qiLsFr0=vr0=vr0dc8meaabaqaciaacaGaaeqabaqabeGadaaakeaacuWGWbaCgaGhamaaBaaaleaacqWGPbqAaeqaaaaa@2FC1@ = (*p*_*i*1_, …, *p*_*iq*_) denote the phase of g˜i
 MathType@MTEF@5@5@+=feaafiart1ev1aaatCvAUfKttLearuWrP9MDH5MBPbIqV92AaeXatLxBI9gBaebbnrfifHhDYfgasaacH8akY=wiFfYdH8Gipec8Eeeu0xXdbba9frFj0=OqFfea0dXdd9vqai=hGuQ8kuc9pgc9s8qqaq=dirpe0xb9q8qiLsFr0=vr0=vr0dc8meaabaqaciaacaGaaeqabaqabeGadaaakeaacuWGNbWzgaGhamaaBaaaleaacqWGPbqAaeqaaaaa@2FAF@. Given phase p˜i
 MathType@MTEF@5@5@+=feaafiart1ev1aaatCvAUfKttLearuWrP9MDH5MBPbIqV92AaeXatLxBI9gBaebbnrfifHhDYfgasaacH8akY=wiFfYdH8Gipec8Eeeu0xXdbba9frFj0=OqFfea0dXdd9vqai=hGuQ8kuc9pgc9s8qqaq=dirpe0xb9q8qiLsFr0=vr0=vr0dc8meaabaqaciaacaGaaeqabaqabeGadaaakeaacuWGWbaCgaGhamaaBaaaleaacqWGPbqAaeqaaaaa@2FC1@, genotype g˜i
 MathType@MTEF@5@5@+=feaafiart1ev1aaatCvAUfKttLearuWrP9MDH5MBPbIqV92AaeXatLxBI9gBaebbnrfifHhDYfgasaacH8akY=wiFfYdH8Gipec8Eeeu0xXdbba9frFj0=OqFfea0dXdd9vqai=hGuQ8kuc9pgc9s8qqaq=dirpe0xb9q8qiLsFr0=vr0=vr0dc8meaabaqaciaacaGaaeqabaqabeGadaaakeaacuWGNbWzgaGhamaaBaaaleaacqWGPbqAaeqaaaaa@2FAF@ uniquely determines a diplotype (a pair of compatible haplotypes), *H*_*i *_= (*H*_*i*1_, *H*_*i*2_), i.e. g˜i
 MathType@MTEF@5@5@+=feaafiart1ev1aaatCvAUfKttLearuWrP9MDH5MBPbIqV92AaeXatLxBI9gBaebbnrfifHhDYfgasaacH8akY=wiFfYdH8Gipec8Eeeu0xXdbba9frFj0=OqFfea0dXdd9vqai=hGuQ8kuc9pgc9s8qqaq=dirpe0xb9q8qiLsFr0=vr0=vr0dc8meaabaqaciaacaGaaeqabaqabeGadaaakeaacuWGNbWzgaGhamaaBaaaleaacqWGPbqAaeqaaaaa@2FAF@|p˜i
 MathType@MTEF@5@5@+=feaafiart1ev1aaatCvAUfKttLearuWrP9MDH5MBPbIqV92AaeXatLxBI9gBaebbnrfifHhDYfgasaacH8akY=wiFfYdH8Gipec8Eeeu0xXdbba9frFj0=OqFfea0dXdd9vqai=hGuQ8kuc9pgc9s8qqaq=dirpe0xb9q8qiLsFr0=vr0=vr0dc8meaabaqaciaacaGaaeqabaqabeGadaaakeaacuWGWbaCgaGhamaaBaaaleaacqWGPbqAaeqaaaaa@2FC1@ = (*H*_*i*1_, *H*_*i*2_). Therefore, for a genotype with *m *heterozygous loci, there are 2^*m*-1^possible resolutions for phase and diplotypes. Let θ˜
 MathType@MTEF@5@5@+=feaafiart1ev1aaatCvAUfKttLearuWrP9MDH5MBPbIqV92AaeXatLxBI9gBaebbnrfifHhDYfgasaacH8akY=wiFfYdH8Gipec8Eeeu0xXdbba9frFj0=OqFfea0dXdd9vqai=hGuQ8kuc9pgc9s8qqaq=dirpe0xb9q8qiLsFr0=vr0=vr0dc8meaabaqaciaacaGaaeqabaqabeGadaaakeaaiiGacuWF4oqCgaGhaaaa@2E8E@ = (*θ*_1_, *θ*_2_, …, *θ*_*T*_) denote population haplotype frequencies where *T *is the total number of haplotypes.

All methods compared in this paper use the maximum likelihood approach or its variation. They all shared the same likelihood function of haplotype frequency θ˜
 MathType@MTEF@5@5@+=feaafiart1ev1aaatCvAUfKttLearuWrP9MDH5MBPbIqV92AaeXatLxBI9gBaebbnrfifHhDYfgasaacH8akY=wiFfYdH8Gipec8Eeeu0xXdbba9frFj0=OqFfea0dXdd9vqai=hGuQ8kuc9pgc9s8qqaq=dirpe0xb9q8qiLsFr0=vr0=vr0dc8meaabaqaciaacaGaaeqabaqabeGadaaakeaaiiGacuWF4oqCgaGhaaaa@2E8E@ = (*θ*_1_, *θ*_2_, …, *θ*_*T*_), which can be written as

L(θ˜)=∏i=1nf(g˜i|θ˜)=∏i=1n∑p˜if(g˜i|p˜i,θ˜)f(p˜i)=∏i=1n∑p˜if(Hi1|θ˜)f(Hi2|θ˜)f(p˜i),     [1]
 MathType@MTEF@5@5@+=feaafiart1ev1aaatCvAUfKttLearuWrP9MDH5MBPbIqV92AaeXatLxBI9gBaebbnrfifHhDYfgasaacH8akY=wiFfYdH8Gipec8Eeeu0xXdbba9frFj0=OqFfea0dXdd9vqai=hGuQ8kuc9pgc9s8qqaq=dirpe0xb9q8qiLsFr0=vr0=vr0dc8meaabaqaciaacaGaaeqabaqabeGadaaakeaacqWGmbatcqGGOaakiiGacuWF4oqCgaGhaiabcMcaPiabg2da9maarahabaGaemOzayMaeiikaGIafm4zaCMba4badaWgaaWcbaGaemyAaKgabeaakiabcYha8jqb=H7aXzaaEaGaeiykaKIaeyypa0ZaaebCaeaadaaeqbqaaiabdAgaMjabcIcaOiqbdEgaNzaaEaWaaSbaaSqaaiabdMgaPbqabaGccqGG8baFcuWGWbaCgaGhamaaBaaaleaacqWGPbqAaeqaaOGaeiilaWIaf8hUdeNba4bacqGGPaqkcqWGMbGzcqGGOaakcuWGWbaCgaGhamaaBaaaleaacqWGPbqAaeqaaOGaeiykaKIaeyypa0daleaacuWGWbaCgaGhamaaBaaameaacqWGPbqAaeqaaaWcbeqdcqGHris5aaWcbaGaemyAaKMaeyypa0JaeGymaedabaGaemOBa4ganiabg+GivdaaleaacqWGPbqAcqGH9aqpcqaIXaqmaeaacqWGUbGBa0Gaey4dIunakmaarahabaWaaabuaeaacqWGMbGzcqGGOaakcqWGibasdaWgaaWcbaGaemyAaKMaeGymaedabeaakiabcYha8jqb=H7aXzaaEaGaeiykaKIaemOzayMaeiikaGIaemisaG0aaSbaaSqaaiabdMgaPjabikdaYaqabaGccqGG8baFcuWF4oqCgaGhaiabcMcaPiabdAgaMjabcIcaOiqbdchaWzaaEaWaaSbaaSqaaiabdMgaPbqabaGccqGGPaqkaSqaaiqbdchaWzaaEaWaaSbaaWqaaiabdMgaPbqabaaaleqaniabggHiLdaaleaacqWGPbqAcqGH9aqpcqaIXaqmaeaacqWGUbGBa0Gaey4dIunakiabcYcaSiaaxMaacaWLjaGaei4waSLaeGymaeJaeiyxa0faaa@9067@

where *f*(*H*_*ij *_|θ˜
 MathType@MTEF@5@5@+=feaafiart1ev1aaatCvAUfKttLearuWrP9MDH5MBPbIqV92AaeXatLxBI9gBaebbnrfifHhDYfgasaacH8akY=wiFfYdH8Gipec8Eeeu0xXdbba9frFj0=OqFfea0dXdd9vqai=hGuQ8kuc9pgc9s8qqaq=dirpe0xb9q8qiLsFr0=vr0=vr0dc8meaabaqaciaacaGaaeqabaqabeGadaaakeaaiiGacuWF4oqCgaGhaaaa@2E8E@) is the probability of haplotype *H*_*ij *_given the population's haplotype frequencies; *f*(p˜i
 MathType@MTEF@5@5@+=feaafiart1ev1aaatCvAUfKttLearuWrP9MDH5MBPbIqV92AaeXatLxBI9gBaebbnrfifHhDYfgasaacH8akY=wiFfYdH8Gipec8Eeeu0xXdbba9frFj0=OqFfea0dXdd9vqai=hGuQ8kuc9pgc9s8qqaq=dirpe0xb9q8qiLsFr0=vr0=vr0dc8meaabaqaciaacaGaaeqabaqabeGadaaakeaacuWGWbaCgaGhamaaBaaaleaacqWGPbqAaeqaaaaa@2FC1@) is the prior probability of phase.

### Empirical Methods

The estimation-maximization (EM) algorithm was used to obtain maximum likelihood estimates of haplotype frequencies, θ˜
 MathType@MTEF@5@5@+=feaafiart1ev1aaatCvAUfKttLearuWrP9MDH5MBPbIqV92AaeXatLxBI9gBaebbnrfifHhDYfgasaacH8akY=wiFfYdH8Gipec8Eeeu0xXdbba9frFj0=OqFfea0dXdd9vqai=hGuQ8kuc9pgc9s8qqaq=dirpe0xb9q8qiLsFr0=vr0=vr0dc8meaabaqaciaacaGaaeqabaqabeGadaaakeaaiiGacuWF4oqCgaGhaaaa@2E8E@[11]. To avoid trapping in a local maximum, the programs implementing EM algorithm require multiple initial values to ensure the global maximum. Excoffier and Slatkin [11] used bootstrapping to estimate standard errors of estimates of haplotype frequencies, and implemented the method in ARLEQIN. Qin et al. [14] implemented Louis' method [36] and implemented the method in PL-EM. Applying estimation equation technique, Li et al. [15] efficiently estimated the haplotype frequencies and their standard errors and implemented the method in HPlus.

### Bayesian methods

Different from the empirical approaches described above, the model-based Bayesian method [16] further assumes that haplotypes are coalescent so future-sampled individual haplotypes *H*_*i *_is assumed to be more similar to the previously sampled haplotypes, *H*_-*i *_[37]. This Bayesian method was implemented in PHASE software program. Another Bayesian method [19] assumes that prior distribution of haplotype frequency θ˜
 MathType@MTEF@5@5@+=feaafiart1ev1aaatCvAUfKttLearuWrP9MDH5MBPbIqV92AaeXatLxBI9gBaebbnrfifHhDYfgasaacH8akY=wiFfYdH8Gipec8Eeeu0xXdbba9frFj0=OqFfea0dXdd9vqai=hGuQ8kuc9pgc9s8qqaq=dirpe0xb9q8qiLsFr0=vr0=vr0dc8meaabaqaciaacaGaaeqabaqabeGadaaakeaaiiGacuWF4oqCgaGhaaaa@2E8E@ follows a Dirichlet distribution with hyperparameter β˜
 MathType@MTEF@5@5@+=feaafiart1ev1aaatCvAUfKttLearuWrP9MDH5MBPbIqV92AaeXatLxBI9gBaebbnrfifHhDYfgasaacH8akY=wiFfYdH8Gipec8Eeeu0xXdbba9frFj0=OqFfea0dXdd9vqai=hGuQ8kuc9pgc9s8qqaq=dirpe0xb9q8qiLsFr0=vr0=vr0dc8meaabaqaciaacaGaaeqabaqabeGadaaakeaaiiGacuWFYoGygaGhaaaa@2E79@ = (*β*_1_, …, *β*_*T*_. Using Gibbs sampling algorithm, Niu et al. sampled a pair of compatible haplotypes for each individual and estimate the haplotype frequencies, and this method was implemented in HAPLOTYPER.

### Comparison Measurements

Accurately estimating haplotype frequencies and inferring individual haplotypes are both critical in assessing haplotype association with disease phenotypes [30-34]. Here we consider two measures to evaluate the accuracy of haplotype frequency estimates and the inferred individual haplotypes. The first measure is the similarity index [11] defined as I(θ^˜;θ˜)=1−0.5∑j=1T|θ^j−θj|
 MathType@MTEF@5@5@+=feaafiart1ev1aaatCvAUfKttLearuWrP9MDH5MBPbIqV92AaeXatLxBI9gBaebbnrfifHhDYfgasaacH8akY=wiFfYdH8Gipec8Eeeu0xXdbba9frFj0=OqFfea0dXdd9vqai=hGuQ8kuc9pgc9s8qqaq=dirpe0xb9q8qiLsFr0=vr0=vr0dc8meaabaqaciaacaGaaeqabaqabeGadaaakeaacqWGjbqscqGGOaakiiGacuWF4oqCgaqcgaGhaiabcUda7iqb=H7aXzaaEaGaeiykaKIaeyypa0JaeGymaeJaeyOeI0IaeGimaaJaeiOla4IaeGynauZaaabCaeaadaabdaqaaiqb=H7aXzaajaWaaSbaaSqaaiabdQgaQbqabaGccqGHsislcqWF4oqCdaWgaaWcbaGaemOAaOgabeaaaOGaay5bSlaawIa7aaWcbaGaemOAaOMaeyypa0JaeGymaedabaGaemivaqfaniabggHiLdaaaa@4B58@, where *θ*_*j *_and θ^j
 MathType@MTEF@5@5@+=feaafiart1ev1aaatCvAUfKttLearuWrP9MDH5MBPbIqV92AaeXatLxBI9gBaebbnrfifHhDYfgasaacH8akY=wiFfYdH8Gipec8Eeeu0xXdbba9frFj0=OqFfea0dXdd9vqai=hGuQ8kuc9pgc9s8qqaq=dirpe0xb9q8qiLsFr0=vr0=vr0dc8meaabaqaciaacaGaaeqabaqabeGadaaakeaaiiGacuWF4oqCgaqcamaaBaaaleaacqWGQbGAaeqaaaaa@3002@ are the true and the estimated frequency of the *j*th haplotype, to measure the overall similarity between the estimated and the sample haplotype frequencies and the value of the similarity index ranges from zero to one. The second measure is the prediction rate that measures the percent of correct predictions for all haplotypes from their genotypes compared to the sampled haplotypes. Since HAPLOTYPER gives only one pair of compatible haplotypes for each individual, we calculated the prediction rate based on the best prediction for each individual. The prediction rate weighted by the posterior probability of a pair of inferred haplotypes was also used to evaluate other programs. The results are similar between the two prediction rates. Running time is recorded to measure the computational efficiency of the implemented algorithms. All computer programs were run under their default or recommended settings on computer with a dual Pentium III 800 MHz with 2 GB RAM.

## Results

### Genotype data on the X-chromosome from the International HapMap project

All four programs inferred haplotypes with high accuracy from the genotypes on the X-chromosome in the 500 selected European haplotype blocks, but HAPLOTYPER failed to resolve any results in 18 blocks and PHASE performed poorly in one of the haplotype blocks with similarity index of 0.29 and prediction rate of 0.28. The mean similarity index and the mean prediction rate are 0.99 and the medians are 1.0 for all programs (Table 1). The standard deviation of the similarity index is 0.029, 0.024, 0.040, and 0.24 and the range is (0.73, 1.0), (0.83, 1.0), (0.29, 1.0), and (0.73, 1.0) for PL-EM, HPlus, PHASE, and HAPLOTYPER, respectively. The standard deviation of the prediction rate is 0.029, 0.025, 0.040, and 0.25, respectively, and the range is (0.73, 1.0), (0.83, 1.0), (0.28, 1.0), and (0.73, 1.0) for PL-EM, HPlus, PHASE, and HAPLOTYPER, respectively. The running time is 98, 20, 9935, and 422 seconds for PL-EM, HPlus, PHASE, and HAPLOTYPER, respectively.

**Table 1:** Performances of haplotyping methods on analyzing 500 randomly selected haplotype blocks of the 30 European mothers' genotypes on X-chromosome from the HapMap data.

**Performances**	**Empirical Method**		**Bayesian Method**	
	
	**PL-EM**	**HPlus**	**PHASE**	HAPLOTYPER*
**Similarity Index**				
Mean	0.989	0.990	0.986	0.991
Median	1.0	1.0	1.0	1.0
Standard deviation	0.029	0.024	0.040	0.024
Range	(0.733, 1.0)	(0.833, 1.0)	(0.292,1.0)	(0.733,1.0)

**Prediction Rate**				
Mean	0.989	0.990	0.990	0.991
Median	1.0	1.0	1.0	1.0
Standard deviation	0.029	0.025	0.040	0.025
Range	(0.733, 1.0)	(0.833, 1.0)	(0.283, 1.0)	(0.733, 1.0)

**Running Time **(in second)	98	20	9935	422

*: HAPLOTYPER failed to resolve 18 of the 500 haplotype blocks.

All four programs performed similarly on the 500 African haplotype blocks as they did on the 500 European haplotype blocks (Table 2), except that HAPLOTYPER failed to converge in 2 blocks. Since African populations have shorter blocks than European populations, all programs required less running time; PL-EM, HPlus, PHASE, and HAPLOTYPER took 15, 7, 1174, and 37 seconds, respectively.

**Table 2:** Performances of haplotyping programs on analyzing 500 randomly selected haplotype blocks of the 30 African mothers' genotypes on X-chromosome from the HapMap data.

**Performances**	**Empirical Method**		**Bayesian Method**	
	
	**PL-EM**	**HPlus**	**PHASE**	HAPLOTYPER*
**Similarity Index**				
Mean	0.988	0.988	0.987	0.998
Median	1.0	1.0	1.0	1.0
Standard deviation	0.025	0.024	0.027	0.025
Range	(0.833, 1.0)	(0.833, 1.0)	(0.689,1.0)	(0.833,1.0)

**Prediction Rate**				
Mean	0.987	0.987	0.987	0.987
Median	1.0	1.0	1.0	1.0
Standard deviation	0.027	0.027	0.030	0.027
Range	(0.800, 1.0)	(0.800, 1.0)	(0.683, 1.0)	(0.800, 1.0)

**Running Time **(in second)	15	7	1174	37

*: HAPLOTYPER failed to resolve 2 of the 500 haplotype blocks.

**Figure 1:**
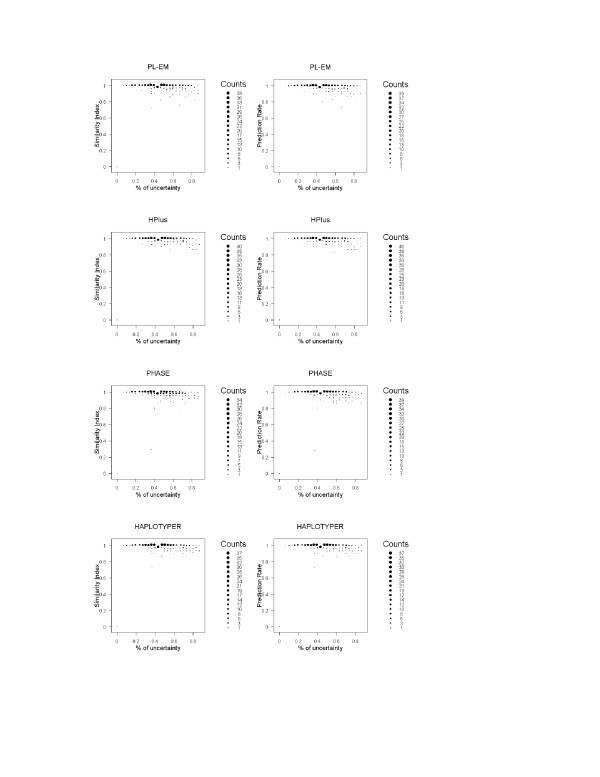
**The relationship between the performances of haplotyping methods and the percentage of individuals with uncertainty haplotypes**. The plots illustrate for the performances (in similarity Index and Prediction Rate) of empirical methods (PL-EM and HPlus) and Bayesian methods (PHASE and HAPLOTYPER) on analyzing the 500 randomly selected haplotype blocks of the 30 European mothers' genotypes on X-chromosome.

**Figure 2:**
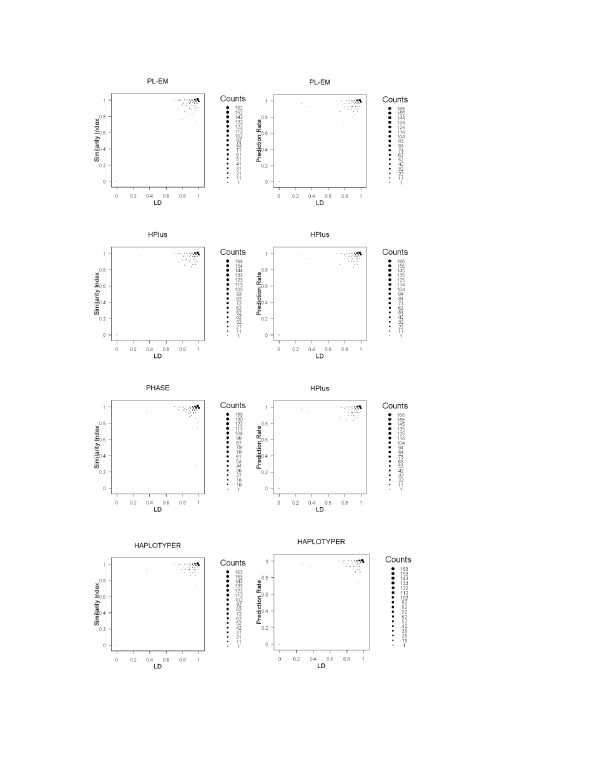
**The relationship between the performances of haplotyping methods and the linkage disequilibrium (LD) of the haplotypes within blocks**. The plots illustrate for the performances (in similarity Index and Prediction Rate) of empirical methods (PL-EM and HPlus) and Bayesian methods (PHASE and HAPLOTYPER) on analyzing the 500 randomly selected haplotype blocks of the 30 European mothers' genotypes on X-chromosome.

In general, the performances of all programs are affected by the percentage of heterozygous individuals, since heterozygosis at multiple loci indicates the uncertainty of individual haplotypes. To investigate the impact of this factor, we examined its relationship with similarity index and prediction rate in analyzing the 500 European haplotype blocks (Figure 1). It appears that all programs tended to perform better for the data with a lower percentage of uncertainty. Even with high percentage of uncertainty in the genotype data, all programs still performed with high accuracy. The other impact factor is the LD of SNPs in the blocks that had been investigated in recent paper [23]. Figure 2 shows the relation between performances with the LD of haplotype blocks. Since our focus is to evaluate program performance on haplotype blocks, SNPs are in high LD within blocks. With high LD, all programs perform well, except PHASE, which had a poor performance on one block. Multi-locus LD is measured using the formulation derived in the paper [38].

### Simulated Data

In the first set of simulations, we randomly selected three series of SNPs with low LD. For each selected series of SNPs, we estimated frequencies from the 60 haplotypes of the 30 European mothers. Based on these frequencies, we randomly drew haplotypes to form genotypes of individuals. The number of individuals was 100, 150, 200, 250, and 300, respectively. For a given sample size, we then generated 100 replicated data sets and analyzed each data set using all four programs. Table 3 shows the average performance of each program over 100 replicates. The similarity index and prediction rate from analyzing the original data are presented in the first row of each block. It is clear that PHASE is superior to the other programs with respect to performance indices for a small sample size, but when the sample size increases, the other programs, especially PL-EM and HPlus, performed as well as PHASE and sometimes (e.g. in the second selected block) outperformed it on the prediction rate.

**Table 3:** Performances of haplotyping programs on simulated data based on some selected genotypes from the 30 European mothers on X-chromosome from the HapMap data.

**#SNP**	**Sample size**	**Similarity Index**				**Prediction Rate**				**Average Running Time (in seconds)**			
		
		**Empirical**		**Bayesian**		**Empirical**		**Bayesian**		**Empirical**		**Bayesian**	
		
		**PL-EM**	**HPlus**	**PHASE**	**HAPLO**^+^	**PL-EM**	**HPlus**	**PHASE**	**HAPLO**^+^	**PL-EM**	**HPlus**	**PHASE**	**HAPLO**^+^
13	30*	*0.783*	*0.776*	*0.822*	*0.683*	*0.767*	*0.767*	*0.800*	*0.633*				
	100	0.969	0.972	0.985	0.979	0.966	0.970	0.977	0.978	0.64	0.16	53.26	2.61
	150	0.981	0.982	0.989	0.981	0.973	0.978	0.989	0.980	0.69	0.23	88.57	2.64
	200	0.986	0.987	0.993	0.984	0.985	0.986	0.992	0.982	0.86	0.30	114.88	5.25
	250	0.988	0.989	0.994	0.979	0.986	0.987	0.992	0.977	0.92	0.38	157.55	6.45
	300	0.927	0.992	0.996	0.984	0.991	0.992	0.992	0.982	0.89	0.40	194.63	7.77
													
16	30*	*0.774*	*0.807*	*0.904*	*0.733*	*0.700*	*0.733*	*0.800*	*0.667*				
	100	0.931	0.933	0.945	0.930	0.897	0.901	0.893	0.905	3.38	1.34	108.74	3.31
	150	0.948	0.951	0.958	0.943	0.909	0.913	0.894	0.919	12.02	1.83	180.08	4.78
	200	0.961	0.963	0.967	0.955	0.923	0.926	0.907	0.932	34.81	2.21	249.74	6.06
	250	0.968	0.968	0.970	0.956	0.930	0.931	0.912	0.934	61.47	2.46	332.92	7.27
	300	0.956	0.971	0.972	0.956	0.928	0.928	0.895	0.932	64.17	2.85	421.54	8.67
													
12	30*	*0.554*	*0.597*	*0.614*	*0.600*	*0.533*	*0.567*	*0.567*	*0.567*				
	100	0.908	0.918	0.925	0.913	0.866	0.873	0.859	0.884	1.46	0.36	76.49	3.49
	150	0.940	0.943	0.945	0.933	0.896	0.899	0.862	0.899	1.52	0.49	127.48	5.08
	200	0.956	0.958	0.957	0.938	0.904	0.906	0.880	0.901	1.61	0.55	173.51	6.61
	250	0.964	0.964	0.963	0.944	0.915	0.917	0.897	0.909	1.64	0.69	202.33	8.85
	300	0.971	0.971	0.969	0.948	0.921	0.921	0.893	0.914	1.67	0.75	278.34	10.13

*: Analysis results of the 30 mothers' genotypes on X-chromosome from the HapMap data

^+^: HAPLO is short for HAPLOTYPER

We also used Hudson's coalescent program to generate phase-resolved haplotype data. We generated data sets with a mutation rate of 4 (= 4N_e_*θ*) and sample sizes of 100, 150, 200, and 250, respectively. For each sample size, we repeated simulations 100 times. Table 4 lists the average performance indices for each program and sample size. For all sample sizes, PHASE consistently performed better than all other programs with respect to both similarity index and prediction rate. This result supports the notion that when the modeling assumption is valid, PHASE is more efficient than other methods (empirical Bayesian or empirical methods). However, it is important to note that the differences between PHASE and others become less marked with larger sample sizes. This result was expected because the gain by PHASE due to the coalescent assumption diminishes and the likelihood methods approach their full efficiency with increased sample sizes. We also conducted simulation studies with different coalescent model parameters, and the results (not shown) are largely comparable to those shown in Table 4.

**Table 4:** Performances of haplotyping programs on simulated data based on a coalescence model with mutation rate of 4 (= 4N_e_*θ*).

**Average #SNP**	**Sample size**	**Similarity Index**				**Prediction Rate**				**Average Running Time (in seconds)**			
		
		**Empirical**		**Bayesian**		**Empirical**		**Bayesian**		**Empirical**		**Bayesian**	
		
		**PL-EM**	**Hplus**	**PHASE**	**HAPLO**^+^	**PL-EM**	**HPlus**	**PHASE**	**HAPLO**^+^	**PL-EM**	**HPlus**	**PHASE**	**HAPLO**^+^
25	100	0.943	0.947	0.982	0.976	0.941	0.945	0.981	0.976	1.39	0.18	122.89	2.52
25	150	0.955	0.960	0.988	0.986	0.952	0.957	0.988	0.986	2.56	0.23	185.86	3.09
26	200	0.967	0.971	0.991	0.988	0.964	0.968	0.988	0.988	5.51	0.29	283.28	4.36
29	250	0.974	0.977	0.992	0.986	0.973	0.976	0.993	0.980	12.37	0.36	429.03	5.75

^+^: HAPLO is short for HAPLOTYPER

In both Tables 3 and 4, average running times are recorded on the far right for comparison purposes. For all simulations, PHASE requires much more computational time than others and HPlus requires the least computational time among the four.

## Discussion

The key difference between the Bayesian and empirical methods compared in this paper is the use of priors (the approximate coalescent prior by PHASE and the Dirichlet prior by HAPLOTYPER). If the prior approximates real haplotype data, Bayesian methods gain some efficiency using the prior. On the other hand, efficiency may be lost because of a wrong prior. The influence of the prior is non-negligible when the sample size is small. In this case, because the real haplotypes tend to coalesce, PHASE using the approximate coalescent prior is likely to produce more efficient estimates, and HAPLOTYPER using the Dirichlet prior may produce less efficient estimates than the empirical methods. This phenomenon was observed when inferring haplotypes from the simulated genotypes using Hudson's coalescent program [25]. However, the superior performance of PHASE diminishes when the sample size increases (Table 4). For genotype data with low LD, PHASE using the approximate coalescent prior would gain some efficiency when the sample size is small, such as 30, which we investigated here, and 104, which Marroni et al. [23] investigated. However, when the sample increases to 150 or larger, PL-EM and HPlus implementing empirical methods can perform as well as PHASE (Table 3).

Recently, Kimmel and Shamir [39] developed a new likelihood method to infer haplotypes and identify haplotype blocks. Their likelihood uses not only the parameters of haplotype frequencies but also the parameters of the probability of observing a variant allele in each locus and each haplotype. Using the EM algorithm, they estimated haplotype frequencies. It deserves debate whether this new likelihood is better than the one used in most methods. In terms of performance, Kimmel and Shamir [39] claimed that PHASE performed slightly better using its default setting and was a hundred times slower than GERBIL implementing the new likelihood method. Their results comparing PHASE and the empirical methods are similar to ours.

## Conclusion

The recent advent of genotyping technologies is rapidly transforming genetic association studies by providing more SNPs (more than 500,000 SNPs) on arrays and by reducing the cost of genotyping individual samples (around $500~1000 per sample). The next-generation genome wide studies will likely use several hundreds or thousands of SNPs on hundreds or thousands of individuals. To gain both statistical and computational efficiency, haplotype-based analyses will be increasingly used, especially for those regions with high LD. With such massive data, as we show in this paper, the empirical methods such as EM and EE can infer haplotypes as accurately as a time-consuming method, such as the model-based Bayesian method that PHASE implement, and they can be easily incorporated into downstream haplotype-based analyses. The empirical methods had already been used in many haplotype-based association methods [32,34,40].

## Competing interests

The author(s) declare that they have no competing interests.

## Authors' contributions

SSL did the study design, directed the analyses, and drafted the manuscript. JJC ran all the analyses and simulations. LPZ contributed to the study design and the draft of the manuscript. All authors approved the final manuscript.
